# Tensions between the Professions: How Integrated Care Can Benefit from Better Understanding and Managing Conflicting Perspectives and Demands

**DOI:** 10.5334/ijic.7570

**Published:** 2023-05-25

**Authors:** Malte Haring, Felix Freigang, Martin Gersch, Volker Amelung

**Affiliations:** 1 Hannover Medical School, Institute of Epidemiology, Social Medicine and Health System Research, Carl-Neuberg-Str. 1, 30625 Hannover, Germany; 2 inav –Private Institute for Applied Health Services Research GmbH, Schiffbauerdamm 12, 10117 Berlin, Germany; 3 Freie Universität Berlin, Department of Information Systems, Garystr. 21, 14195 Berlin, Germany

**Keywords:** tensions, healthcare system, integrated care, workforce

## Abstract

In the transition from fragmented to integrated care, tensions are inevitable. Contradictions between the actors of the different professions involved can have negative but also positive effects on change processes in the healthcare system. This is especially true for integrated care, where collaboration among the workforce is pivotal. Therefore, efforts should not be made to avoid tensions from the outset, if this is at all possible, but to deal with them constructively. The attention of leading actors must be increased to recognize, analyse, and successfully manage tensions. The creative potential of tensions can be harnessed to successfully implement integrated care and engage the diverse workforce.

## Abstract (de)

Beim Übergang von der fragmentierten zur integrierten Versorgung sind Spannungen unvermeidlich. Widersprüchlichkeiten zwischen den Akteuren der verschiedenen an der Versorgung beteiligten Berufsgruppen können sich sowohl negativ als auch positiv auf Veränderungsprozesse im Gesundheitswesen auswirken. Dies gilt insbesondere für die integrierte Versorgung, bei der die Zusammenarbeit zwischen den Arbeitskräften von zentraler Bedeutung ist. Es sollte daher nicht versucht werden, Spannungen von vornherein zu vermeiden, sofern dies überhaupt möglich ist, sondern konstruktiv mit ihnen umzugehen. Die Aufmerksamkeit der verantwortlichen Akteure muss erhöht werden, um Spannungen zu erkennen, zu analysieren und erfolgreich zu bewältigen. Das kreative Potenzial von Spannungen kann genutzt werden, um die integrierte Versorgung erfolgreich umzusetzen und die diversen Arbeitskräfte in diese einzubinden.

## Introduction

**Figure 1:**
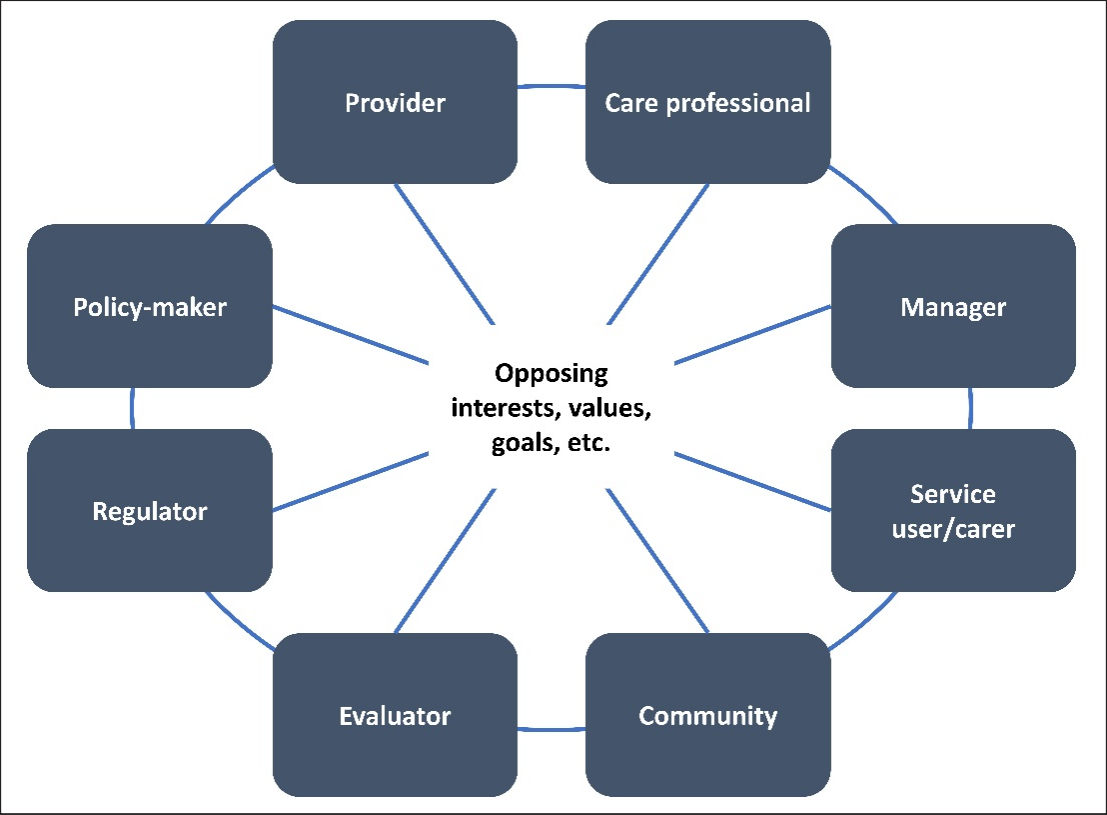
(Opposing) perspectives that determine integrated care based on Shaw et al., 2011 [4].

Integrated care refers to “the effort to improve the quality of care for individual patients, service users, and caregivers by ensuring that services are well coordinated around their needs across different care environments” [1, pp. 1–2]. For the realization of integrated care, different professions and disciplines must therefore work together, each bringing their identity and culture to healthcare. These are characterized by divergent, almost fundamentally opposing objectives and values. This integration of different professional groups, their motivations, ethics, and perspectives shapes (integrated) care and influences its development (Figure 1) [2,3].

At the same time, the implementation of integrated care faces the fundamental challenge of achieving the necessary cooperation and coordination between cultures. This requires that the organizations, sectors, and professions involved in care leave their familiar value structures and instead develop a common culture of values. The resulting loss of identity harbours potential for conflict since the workforces involved in care have a strong identification with their professions and the organizational structures that encompass them [3,5]. One symptom, but also the reason for this, can be tensions: The different demands and expectations of the professions involved in the provision of healthcare represent opposing poles that lead to points of friction in the collaboration and ultimately to tensions [6].

### Different expectations and conflicting demands

**Figure 2:**
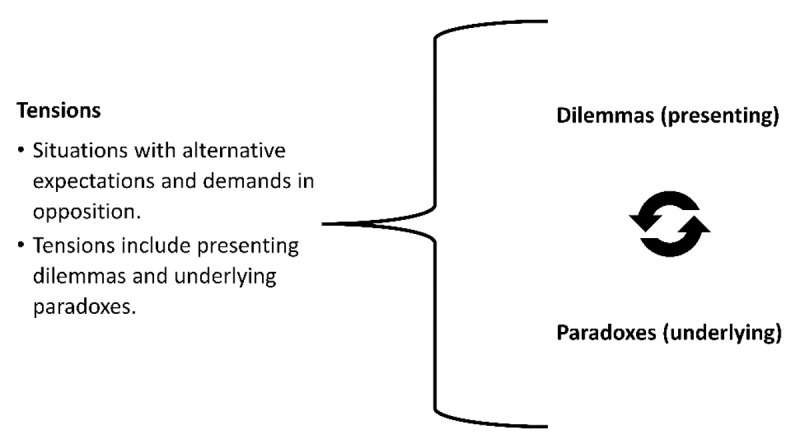
Theory of tensions based on Smith and Lewis, 2022 [7].

Tensions appear in the form of visible dilemmas and as a result of the underlying opposites and contradictions (paradoxes) (Figure 2) [7]: In the context of innovative healthcare, this means, for example, that newly developed approaches conflict with established practices, internal guidelines with external needs, and the actors’ desire for autonomy with standardization – although they are often not openly discussed by those involved in the healthcare system [8].

Tensions are thus both symptoms of change processes, but also have the potential to trigger (re)actions that can have a reinforcing (virtuous cycles) or restraining (vicious cycles) effect on these processes [6,7]. In the negative case, this means that care continuities and processes are massively disrupted by conflicts between the professions and disciplines involved in healthcare, making collaboration and joint goal achievement impossible. In the positive case, tensions can be important drivers for integrated care, resulting in the development of new solutions and progress in the implementation of integrated care. For instance, error reporting systems are a good example of how to deal productively with conflicts in the context of establishing a patient safety culture by integrating the perspectives and convictions of different professional groups and cultures [7,9,10].

This situation of the (unavoidable) emergence of tensions as a consequence of the enforced integration of contradictory elements is intensified by the degree of complexity of the respective form of care. This ranges from bridging single gaps in care (e.g., discharge management in hospitals) to complex systems in which health and social care merge completely (e.g., population health). However, the more complex the care processes and the more perspectives are brought together in this course, the more complex the interests are, and the greater the potential for tensions appears to be – especially when different care areas and sectors, as well as professional groups working there, are involved [2,11].

### You can only cope with what you understand

Therefore, it is important to better understand tensions and contradictions in healthcare and to enable a reflexive approach to their effects. This is particularly true in view of the aging of society and the simultaneous decline in financial and human resources in the healthcare system. These developments increase tensions and thus jeopardize the success of healthcare, healthcare performance, and integrated care [2,12,13].

Entry points are a) a better understanding of causes and the emergence of tensions, b) a better understanding of the characteristics and types of tensions in organizations and change processes, and c) the application of appropriate management strategies for a goal-oriented handling of tensions and the harnessing of activation potentials concerning intended changes driven by the tensions [6,7].

Integrated care should leverage these potentials and consider and implement findings from research on organizational tensions in change processes. In general, the consideration of findings from the management literature represents great potential for the healthcare sector. A paradigm shift is indicated here, in which findings are not transferred unchecked, but reflectively relevant theories and practices are adopted for the development of the healthcare system [14].

## Building understanding of tensions to reduce inhibiting conflicts and increase effectiveness

Smith and Lewis [6] identified and described four main categories for classifying tensions (description here is based on Gersch (2022) with examples from Haring et al. (2022) [15,8]:

- **Organizational tensions** occur when multiple organizational systems are combined and condition competing designs and processes (for example, differentiation vs. standardization [16]),
- **Performing tensions** occur primarily when multiple actors pursue divergent goals in a factually necessary collaboration (for example, quality improvement vs. implementation costs [17]),
- **Learning tensions** involve using and often destroying past practices to create new ones (for example, facilitating externals vs. satisfying internals [18]),
- **Belonging tensions** arise from competing identities with which individuals identify. These occur, among other things, when tasks are attempted to be shifted, reassigned, or changed across established professional boundaries (for example, interdisciplinary work vs. professional autonomy [19]).

**Figure 3:**
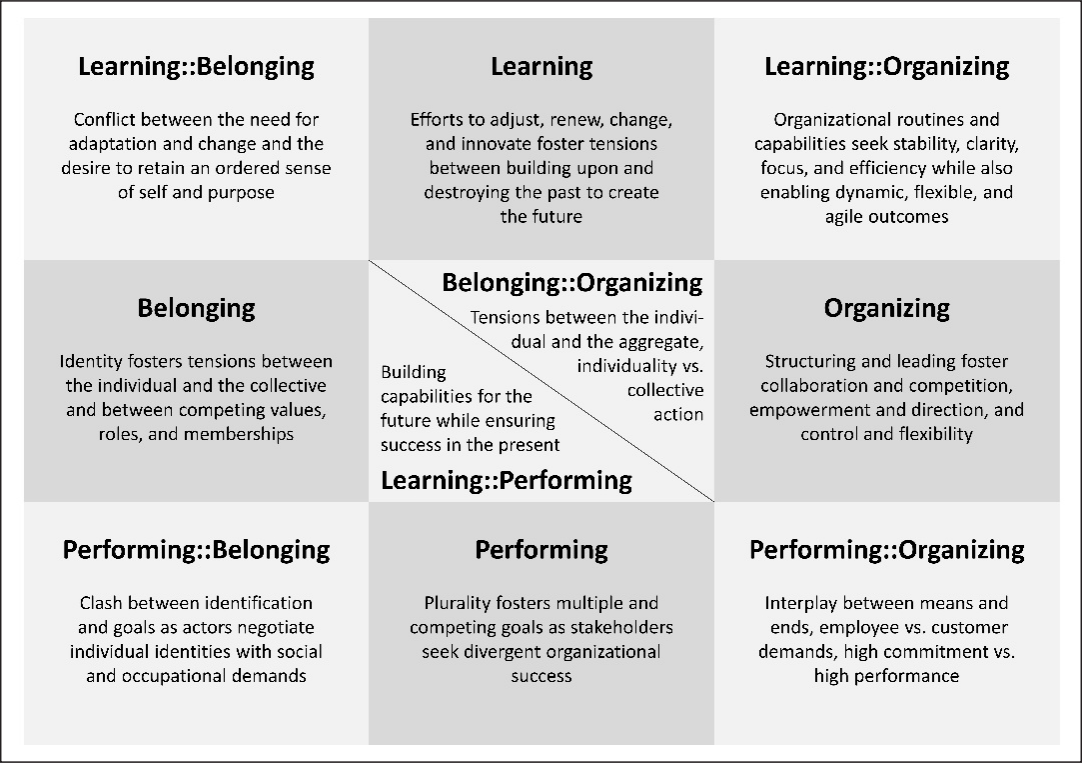
Categories of organizational tension based on Smith and Lewis, 2011 [6].

In addition, there are also six cross-cutting categories that can be used to classify tensions that cannot be assigned to a single category but fall between two different categories. Figure 3 provides an overview of the categories of tensions.

In the context of integrated care and its workforce, all of these categories are relevant, but consideration of the tensions of some categories seems particularly important. Specifically, these include the tensions associated with the categories “Belonging”, “Performing”, and “Performing::Belonging”. Also relevant, but secondary, are the tensions from the categories “Learning” and “Organizing” as well as from the other cross-cutting categories.

### Strategies for managing tensions between the integrated care workforce

Effective management must start here and deal reflexively with the conflict and the underlying causes. Management procedures should be developed based on the classification of the tensions, which have a tension-relieving effect on the prevailing conflict situation. Three basic management strategies are described in the literature and have been shown to promote virtuous cycles and avoid vicious cycles [6,7,16,20,21]:

- **Either-or** (Separate conflicting demands and resolve the conflict by choosing one element over the other),
- **Both-and** (Achieve balance between the conflicting poles and integrate them),
- **More-than** (Develop a new perspective out of the conflict or create a new element that surpasses the existing divergences).

Consequently, effective management of tensions and the dynamics associated with them considers both poles of contradiction and provides answers for dealing with divergent demands. In this way, constructive effects (virtuous cycles) and synergies can be achieved that promote exchange, development, and the positive course of a change process. However, ignoring organizational tensions or opposing poles can also lead to destructive effects (vicious cycles). Reflective and successful management of tensions requires selecting appropriate measures to respond appropriately to the different types and strengths of tensions in the concrete implementation of integrated care [6,7,20,22,23].

Therefore, concrete activities to manage tensions in the context of an integrated care workforce should be done through agile (change) management that also accounts for tensions: Fostering communication among stakeholders, central coordination, and establishing an understanding of the goals and views of the other professional groups involved in healthcare. These reflexive and iterative management activities encompass the required positive feedback culture and can be used to respond to tensions that arise spontaneously and to support the integrated care workforce by recognizing their needs, serving their (conflicting) demands, and creating an environment of trust and collaboration through shared goals and understanding of each other‘s needs and objectives [7,8]. This is particularly relevant considering aging populations, dwindling resources, and the changing demands of collaborative healthcare, in which the diverse profiles and roles of the (multi-) professional groups involved are also changing [2,7].

## Tensions are not necessarily negative – the wrong way of dealing with them is

Since the framework conditions of the healthcare system in general, and of integrated care, in particular, lead us to assume that tensions will always arise in principle, it is insufficient to allow unreflective reaction dynamics or to focus on avoiding and/or overcoming any tensions as quickly as possible. Rather, it is a task of reflective management to orchestrate tensions and use them as motivators for change. This involves allowing a more positive feedback culture in healthcare, expecting tensions from the outset, reflecting on them, and thus also mitigating their destructive effects. With these dynamics, the creative potential of tensions can be harnessed to implement integrated care and engage as well as moderate the diverse workforce and include their perspectives in the process. To this end, the management of tensions must be considered a relevant activity in resource planning for change processes and integrated care projects. In addition, the training of the workforce must already focus on tension-sensitive conflict management and create spaces for the development and expansion of understanding of the perspectives and cultures of other professions involved in care. One possible approach here is the concept of a health campus with a common educational identity for diverse professions.
